# Assessing the Needs and Perspectives of Patients With Asthma and Chronic Obstructive Pulmonary Disease on Patient Web Portals: Focus Group Study

**DOI:** 10.2196/formative.8822

**Published:** 2018-11-22

**Authors:** Esther Metting, Aaltje Jantine Schrage, Janwillem WH Kocks, Robbert Sanderman, Thys van der Molen

**Affiliations:** 1 Groningen Research Institute for Asthma and COPD Department of General Practice and Elderly Care Medicine, University Medical Center Groningen University of Groningen Groningen Netherlands; 2 GZW-Health Psychology–GZW-General University Medical Center Groningen University of Groningen Groningen Netherlands; 3 Department of Psychology, Health & Technology Faculty of Behavioural, Management and Social Sciences University of Twente Enschede Netherlands

**Keywords:** asthma, chronic obstructive pulmonary disease, health care, health literacy, internet, electronic medical record, self-management

## Abstract

**Background:**

As accessibility to the internet has increased in society, many health care organizations have developed patient Web portals (PWPs), which can provide a range of self-management options to improve patient access. However, the available evidence suggests that they are used inefficiently and do not benefit patients with low health literacy. Asthma and chronic obstructive pulmonary disease (COPD) are common chronic diseases that require ongoing self-management. Moreover, patients with COPD are typically older and have lower health literacy.

**Objective:**

This study aimed to obtain and present an overview of patients’ perspectives of PWPs to facilitate the development of a portal that better meets the needs of patients with asthma and COPD.

**Methods:**

We performed a focus group study using semistructured interviews in 3 patient groups from the north of the Netherlands who were recruited through the Dutch Lung Foundation. Each group met 3 times for 2 hours each at a 1-week interval. Data were analyzed with coding software, and patient descriptors were analyzed with nonparametric tests. The consolidated criteria for reporting qualitative research were followed when conducting the study.

**Results:**

We included 29 patients (16/29, 55% male; mean age 65 [SD 10] years) with COPD (n=14), asthma-COPD overlap (n=4), asthma (n=10), or other respiratory disease (n=1). There was a large variation in the internet experience; some patients hardly used the internet (4/29, 14%), whereas others used internet >3 times a week (23/29, 79%). In general, patients were positive about having access to a PWP, considering access to personal medical records as the most important option, though only after discussion with their physician. A medication overview was considered a useful option. We found that communication between health care professionals could be improved if patients could use the PWP to share information with their health care professionals. However, as participants were worried about the language and usability of portals, it was recommended that language should be adapted to the patient level. Another concern was that disease monitoring through Web-based questionnaire use would only be useful if the results were discussed with health care professionals.

**Conclusions:**

Participants were positive about PWPs and considered them a logical step. Today, most patients tend to be better educated and have internet access, while also being more assertive and better informed about their disease. A PWP could support these patients. Our participants also provided practical suggestions for implementation in current and future PWP developments. The next step will be to develop a portal based on these recommendations and assess whether it meets the needs of patients and health care providers.

## Introduction

### Self-Management

Annually, 38 million people worldwide die from noncommunicable diseases caused by unhealthy lifestyles. These diseases are chronic [1], and most are suitable for long-term self-management by self-monitoring, lifestyle changes, and symptom control. The aim of self-management is to improve physical, social, and mental well-being [2]. However, this requires the involvement of patients with their disease, which necessitates a greater understanding of their disease [3]. It has been shown that 60% of Europeans look for health information over the Web and almost 90% of these are satisfied with their findings [4]. Internet use has become increasingly important in health care, with the ever-increasing potential to improve outcomes [5,6]. Many Web-based tools have therefore been developed to support patient self-management, including smartphone apps, information websites, and patient Web portals (PWP).

### Patient Web Portals

A PWP is a secure website provided by a health care provider, which serves as a gateway to services ranging from access to health records to the ability to contact a health care provider or make appointments over the Web [7]. Through apps, PWPs can provide these services that enhance patient involvement in care [8] and can provide tailored and timely information [9] by linking health information to medical records [10]. Many disease-specific portals exist (eg, mental illness and diabetes) [11], but portals have also been developed to present overviews of radiology reports [12] or reconcile medication regimens after hospital discharge [13].

Research into the benefits of PWPs is conflicting. Some research has shown the benefits of PWPs on disease status, patient satisfaction, or self-management, whereas others have shown no change in these parameters. Unfortunately, service accessibility varies significantly from easy to difficult [14]. One systematic review showed that self-management, communication [15], or medication adherence improved in some studies, but that there was no significant change in other studies [16,17]. Another problem is that studies have lacked clear outcome measures for the effect of the PWP [16]. Despite these shortcomings, PWPs have been associated with positive outcomes in the treatment of diabetes and hypertension [18-23] and have been shown to improve self-management and patient-physician communication [11,15]. Indeed, PWPs in psychiatric services can increase feelings of autonomy and improve appointment attendance [24,25], while in patients with osteoporosis, PWPs can improve self-management decisions [26]. However, PWP is known to decline over time, with long-term adherence often being poor [27].

### Digital Divide

The digital divide is the phenomenon where younger and more highly educated patients are more likely to use digital technology compared with their older and less-educated peers [15,27,28]. Health literacy, the ability to acquire, read, and understand health information to make appropriate health decisions [29], also needs to be taken into account when developing a PWP. Health numeracy, which can be unrelated to health literacy, is the ability to understand numeric results (eg, lab results). This is compounded because most people overestimate their numeric skills [30]. These issues have huge implications for the presentation of test results and medication advice [31] in a PWP.

The elderly are less likely to use digital technology because of security concerns and the increased effort needed to learn the technology. Motivation, negative attitudes, and satisfaction are other important predictors of PWP use in this context [32,33]. This is important to take into account because a typical population suffering from chronic obstructive pulmonary disease (COPD) has an average age of 67 years [34]. However, Dutch elderly are experienced internet users. In the Netherlands, 71% of citizens between 65 and 74 years are daily internet users; this is far above the average European internet usage of people between 65 and 74 years, which is 39% [35,36].

Patients from low socioeconomic groups are less likely to have internet experience because of health literacy or financial barriers [11,23,32]. This is important information for this paper because COPD and asthma are more prevalent in low socioeconomic populations [37,38]. Minorities and patients with low socioeconomic and educational status are difficult to reach through a PWP [11,18,32,37]. This is concerning because these groups are most prone to having chronic conditions and poor lifestyle behaviors [38].

When building a PWP, developers must take these difficulties into account [38-42]. A PWP should be accessible, understandable, and easy to use [39], especially for older adults [38] and patients with little or no internet experience [14]. Moreover, organizational commitment is needed to ensure successful implementation [9,43,44], focusing on training health care professionals in the proper use of the PWP [15]. Patients can be encouraged to use the PWP by improving immediacy and personalization of the content [45]. To achieve these aims, end users should advise developers [42].

### Aims

Asthma and COPD are common chronic respiratory illnesses that require ongoing self-management. These patients might be supported by a PWP. In this study, we aimed to evaluate the needs and perspectives of patients with asthma or COPD regarding PWPs to facilitate the development of future PWPs adapted to the needs of end users. Specifically, we evaluated their opinions regarding the daily effects of asthma and COPD, internet and health care use, access to medical records, suitable apps, and the relationship between patient and physician. We followed the consolidated criteria for reporting qualitative research [45].

## Methods

### Study Design

#### Participants

Participants were recruited through a patient organization (the Dutch Lung Foundation). Most of them lived in low socioeconomic areas in the north of the Netherlands. We chose to include these areas because patients with low socioeconomic status are often not included in scientific studies. To develop a PWP for this population, it is essential to also include patients with low social economic status. If the portal is understandable and usable for this group, it will be for all patients. We even picked up patients by car if they did not have transportation possibilities to attend the meetings. All participants signed informed consent. The Ethics Committee of the University Medical Center Groningen deemed that the study was not subject to the requirements of the Dutch legislation on “Medical Research Involving Human Subjects” (M13.139696).

#### Structure of the Focus Group Meetings

**Figure 1:**
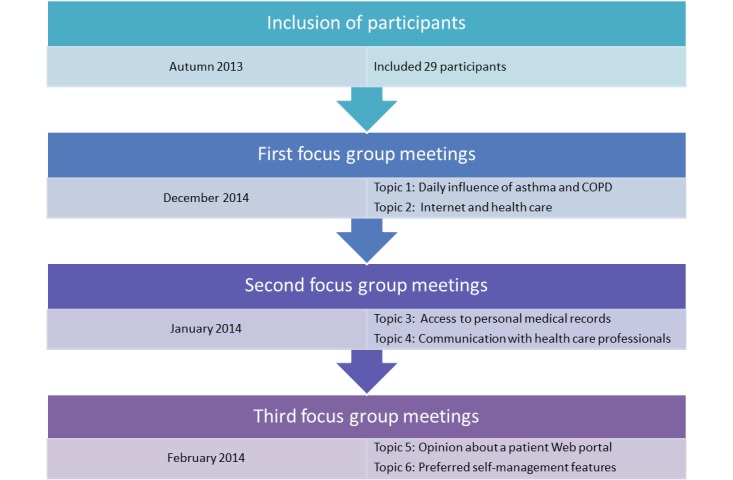
Overview of the focus group meetings and the discussed topics. COPD: chronic obstructive pulmonary disease.

The focus groups were conducted by a psychologist or an epidemiologist trained for that purpose. Participants were placed in 3 groups according to where they lived, and attended 3 meetings at an average weekly interval. Each meeting lasted 2 hours with a 10-minute break half-way through. Meetings took place in 2013 and 2014 at easily accessible locations. All meetings were audiorecorded and videorecorded. Participant involvement was encouraged by providing regular newsletters about the status of the study. Figure 1 provides an overview of the meetings.

#### Focus Group Interview Structure

This is a qualitative study aiming at evaluating the needs and opinions of patients with asthma or COPD. Qualitative studies are exploratory. The aim was to get insight into the needs and opinions of participants. The results were provided by the focus groups, not by individual participants. It was, therefore, not possible to count the opinions of our participants. We can quantify patients’ opinions in future studies with the results of this study as the starting point.

We used semistructured interview schedules covering “Internet and health care,” “Access to personal medical records,” “Patient-physician relationship,” “Features,” and “Self-management.” Videos and PowerPoint slides were used to introduce and explain different topics. We alternated group discussions with individual assignments in which participants had to write their thoughts on post-it notes, which were then used as the starting points for further group discussion.

#### Hypothetical Patient Web Portal Used for the Discussion About Features

Patients in this study had no access to a PWP. Their opinions were based on a hypothetical PWP. The findings will be used to build a PWP in an integrated primary care system for respiratory patients. We discussed commonly provided portal features and used a video with an example of a PWP from a Dutch hospital to enhance the discussion about “Features.” The features presented in this video [46] are shown in Textbox 1.

#### Participant Characteristics Questionnaire

Before the group meetings, participants received a purpose-developed questionnaire that consisted of 12 multiple-choice questions and 3 open questions. This was used to collect information about demographics, internet use, education, and medical history and could be answered on the Web or paper.

### Data Analysis

**Figure 2:**
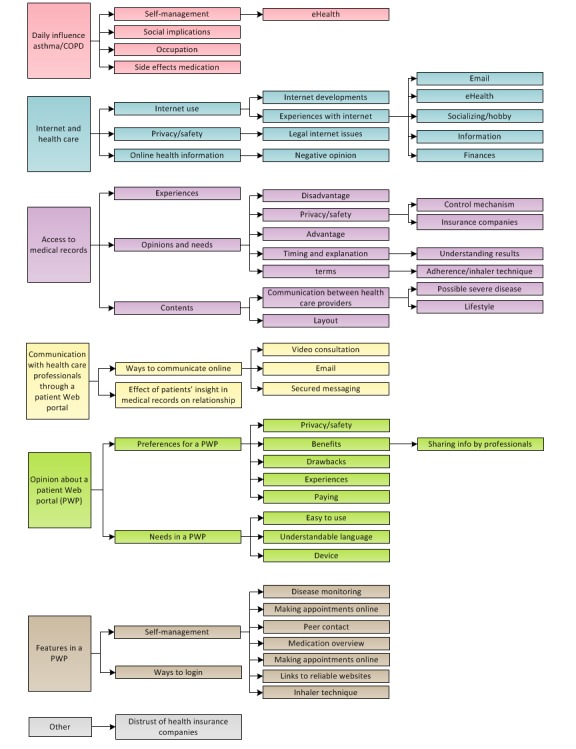
Overview of the different codes. COPD: chronic obstructive pulmonary disease.

All recordings were transcribed verbatim and thematically coded by 2 researchers independently using Kwalitan Version 7. After the decoding procedure, a consensus was reached between the researchers. We used the following 6 thematic codes (Figure 2): (1) daily influence of asthma and COPD; (2) internet and health care; (3) access to personal medical records; (4) communication with health care professionals; (5) opinion about a PWP; and (6) preferred self-management features. We used IBM SPSS Version 22 (IBM Corp, Armonk, NY, USA) for the descriptive analysis. Data are presented as mean and SDs. Differences in characteristics between frequent and infrequent internet users were compared by nonparametric tests.

**Features from the patient Web portal video that were used for the discussion.**

- Logging in
- Web-based access to medical records
- Information about examinations and treatments
- Hospital appointments
- X-ray results
- Laboratory results
- Chatting with other patients
- Asking questions at a secured forum
- Contact with health care provider
- Medication monitoring
- Disease monitoring

## Results

### Summary

The results section provides an overview of demographics and qualitative results. The qualitative results are divided into the main research topics. Textbox 2 summarizes the content of the results section.

### Focus Group Characteristics

We included 29 Dutch-speaking adults, and their characteristics are summarized in Table 1; 23 participants who used the internet >3 times a week were on average younger (mean age 65.2 (SD 8.5) years) than the 6 who used the internet <4 times a week (mean age 74.3 (SD 10.9) years; Kruskal-Wallis test: *P*=.02). All but one participant regularly used email. Several older participants also reported taking computer courses.

### Daily Influence of Asthma and Chronic Obstructive Pulmonary Disease

#### Occupational and Daily Life Restrictions

All participants experienced restrictions in daily life, especially in physical activities:

The list of things you can do gets shorter while the list of things you cannot do gets longer.

Several patients needed to quit working because of asthma or COPD. Differences were described between those with asthma and COPD:

We asthma patients have good times and troubled times. And you [COPD patients] always have bad times.

#### Self-Management Can Improve Symptoms of Asthma and Chronic Obstructive Pulmonary Disease

Planning was also made difficult because symptoms and fatigue can vary from day to day. Patients frequently mentioned the need to plan activities: “It [energy] is like money, you can only spend it once.” Participants also commented on the need to adapt their lifestyles (eg, smoking cessation, regular exercise, or physiotherapy). Typical for patients with asthma and COPD is the need to avoid triggers or other symptom-provoking triggers (eg, fires and barbeques in winters and summers, respectively).

#### Side Effects of Inhaled Medication

Comorbidities were prevalent in our groups, and the medication use was considered important for good self-management. However, many participants reported side effects of the inhaled medication, including stridor, bruises, and cramps. Most patients discussed this with their physicians or pharmacists. Sometimes patients received other medication to reduce the side effects.

#### Social Implications of Asthma and Chronic Obstructive Pulmonary Disease

Asthma and COPD have social implications because they are invisible and the severity varies:

People do not see many signs of illness, but…I have to deal with my chronic condition [daily].

Specific for this patient population is the fact that environmental air can provoke symptoms. Participants explained that they experienced difficulties because others do not understand how allergens like smoke can exacerbate symptoms. They often feel not supported in their avoidance of triggers, which can lead to social isolation because others do not want to adapt their behavior (eg, quit smoking). Moreover, COPD is mostly caused by smoking and others might see the disease as self-inflicted, which leads to stigmatization.

### Internet and Health Care

#### Internet Use and Experience of Patients

The internet was often used to search for information (eg, “If I want to know something, I will look it up”), watch movies, read newspapers, or play games. Others mentioned using Skype, internet banking, Web shops, or second-hand markets. Infrequent internet users were not willing to learn new uses:

The problem is that everything works different…each time you have to put effort in learning again, and I don’t want that.

Some participants had used Web-based health apps, with one using a COPD app provided by their pharmacist; however, he was dissatisfied because he felt that the pharmacist collected his data. Another participant was satisfied with a nutritional app. Some participants valued YouTube movies about inhaler techniques.

**Overview of the topics in the results section (Focus group characteristics). COPD: Chronic Obstructive Pulmonary Disease.**

- Topic 1: Daily influence of asthma and COPD
- Topic 2: Internet and health care
- Topic 3: Access to personal medical records
- Topic 4: Communication with health care professionals
- Topic 5: Opinion about a patient Web portal
- Topic 6: Features in a patient Web portal
- Other

**Table 1:** Patient characteristics of the focus groups participants (N=29) with COPD (n=14), asthma-COPD overlap (n=4), and asthma (n=10).

Characteristic		Total groups		Group I		Group II		Group III	
				Groningen (n=8)		Emmen (n=11)		Assen (n=10)	
Age in years, mean (SD)		67.1 (9.6)		63.8 (9.9)		67.8 (9.7)		68.9 (9.7)	
Gender (male), n (%)		16 (55)		5 (63)		5 (46)		6 (60)	
**Diagnoses, n (%)**									
	Asthma		10 (35)		2 (25)		6 (55)		2 (20)
	COPD^a^		14 (48)		5 (63)		2 (18)		7 (70)
	ACO^b^		4 (14)		1 (13)		2 (18)		1 (10)
	Other		1 (3)		N/A^c^		1 (9)		N/A
**Internet use, n (%)**									
	<4 d/wk		6 (21)		1 (13)		3 (27)		2 (20)
	≥4 d/wk		23 (79)		7 (88)		8 (73)		8 (80)
**Education level, n (%)**									
	Low		9 (31)		2 (25)		3 (27)		4 (40)
	Medium		13 (41)		4 (38)		3 (27)		6 (60)
	High		12 (41)		3 (38)		6 (55)		3 (30)

^a^COPD: chronic obstructive pulmonary disease.

^b^ACO: asthma-COPD overlap.

^c^N/A: not applicable.

#### Patients’ Opinions About Privacy and Safety of the Internet

Several participants were worried about the internet safety and that governmental organizations increasingly rely on the internet (eg, “Sometimes you cannot oversee the overall consequences”). This is, in part, was related to the scandals in the winter of 2014 regarding the safety and usability of these websites in the Netherlands. The government uses a digital system to communicate with citizens, and it was feared that criminals could easily access valuable information like bank account numbers. Participants were also worried that some people could not use websites, especially older people. Others were happy with this development because it makes things easier.

#### Patients’ Experiences and Needs Regarding Web-Based Health Information

One participant searched over the Web for alternative treatments when unsatisfied with her care (eg, “Every prescribed treatment made me sicker. Therefore, I decided to [look] for myself.”), but most searched for health information and information regarding upcoming treatments or examinations. One participant searched for information about Alzheimer’s disease when his father was diagnosed. However, several explained that they did not feel the need to surf the Web if their disease was well controlled. A disadvantage of Web-based information was that unnecessary anxiety and worry could result from the information not being adapted to individuals. It was notable that many had difficulties finding reliable and understandable websites (eg, too many medical terms), which led to some avoiding Web-based information. Others were satisfied with links to reliable websites that were provided by their health care provider.

### Access to Personal Medical Records

Most participants wanted access to their medical records and considered this the most important requirement of a PWP. One even wanted the opportunity to change things in his record. However, some wanted no access (eg, “I know how I feel”).

#### Experiences of Patients Who Already Have Access to Personal Medical Records

Several participants had seen their medical records on paper because they changed general practitioner (GP), were curious, or wanted to compare current and past results:

It surprised me that…when I read it, it was like it was about someone else

Health care providers sometimes doubted whether participants had the right to access their records, and in some cases, refused to provide them; this angered one participant (“This is my data!”).

#### Patients’ Opinions and Needs Regarding Accessible Personal Medical Records

Patients wanted information about prescribed medication and a summary of medical visits, stating that they often had difficulties recalling information provided during consultations:

If I visit a physician I take my wife with me and often, when we get home, I have heard something [different to] my wife.

Web-based records could also be shown by the patient to other health care providers in emergencies. It was emphasized that Web-based information should provide a clear overview of examination results, helping patients become better informed about their disease. In turn, this could help them to prepare for a medical visit and communicate about their disease. Others thought that they might be taken more seriously if they were better informed (eg, “[physicians] need to take patients more serious”).

#### Preferred Content in the Personal Medical Records

##### Crude Assessment of Results

Most participants wanted lab results, reference values, and an explanation, stating “in that way you are well informed,” and emphasizing that results should be presented in lay terms. However, there was recognition of the need to have insight, having physicians first explain the results: “You will get sick and worried if you read [medical terms]!”. Some participants were not interested in this option, feeling sufficiently well informed by their physicians; others wanted psychiatric information to be excluded. One patient tried to commit suicide years ago and did not express any desire to share his experience with other health care professionals because he considered it too personal. There was also a desire to see x-rays, but with the caveat:

…if it takes a few hours to explain what it means, then I don’t want to know.

The groups often mentioned that information takes a long time to be transferred to the GP after attending hospital, meaning that the GP is not always up to date. In these instances, patients could share information with their GP.

##### Consideration of Physician

We discussed whether there was a desire to see if physicians wanted further examinations to exclude severe disease. Most participants wanted this information in the PWP, but to avoid anxiety and worry, only after the examination results and options had been explained (one participant wanted to know immediately, stating “[the] sooner the better”). Several participants felt it would be safer to provide patients with a summary of the findings, not with the consideration of the physician:

I want to know what is wrong with me, not what can possibly be wrong.

It was notable that some wanted both details of any interpretations and the name of the physician, so that they could approach them if they disagree.

##### Lifestyle Advice From the Physician

Some participants considered lifestyle recommendations from their health care professional helpful, even suggesting making these firm requirements to stimulate change. However, others would feel judged or angry (eg*,* “This is how they think about me”), and one even said that such remarks might stop them from going to the physician again.

##### Communication Between Physicians (eg, Referral Letters)

There were comments that patient access could change the way physicians communicate:

He will think: ‘wait a minute, my patient can read this too. I need to make this understandable for my patient’

Some were worried PWPs may make the patient too informed (eg, “What if we [patients] ask many irrelevant questions that have already be considered by the physician?”), whereas others wanted physicians to take patients more seriously. It was recognized that this may change the communication dynamic.

### Communication With Health Care Professionals

#### Ways to Communicate Over the Web With Health Care Professionals Though a Patient Web Portal

Some already communicated with their health care provider through the internet (eg, “mostly after I have visited a specialist I send my GP an email”). One participant explained that it is nice to know that they have the email address, even if it is never used. Some participants have been satisfied when using Skype with health care providers, but most were not familiar with the service and were negative about the possibility of using it for contact. Several disadvantages of Web-based contact were mentioned, with one being that doctors could miss information when communicating through the internet (eg, in face-to-face assessments “you can see how someone is breathing…and what your color is.”). Despite this, most participants welcomed the possibility of Web-based contact to ask health care professionals general questions about asthma or COPD. However, it was felt that Skype meetings should be short and be reserved either to evaluate whether there is an emergency or to conduct routine visits, and only if the patient was comfortable with the method.

#### Effect of Patients’ Access on the Relation With Their Health Care Provider

Participants explained that the internet helps inform patients, which can alter the level of communication with health care professionals:

It will be easier for physicians if you know what they are talking about.

A drawback of the PWP was that physicians might not be able to judge what information to give and what to withhold, the way they might be able to in face-to-face consultations. Physicians should, therefore, be trained on how to deal with assertive and better-informed patients.

### Opinions About a Patient Web Portal

#### Preferences of a Patient Web Portal

##### Opinions About Privacy and Safety

Opinions on privacy and safety varied, with some being worried (eg, “My pulmonologist does not have to see why I have visited the gynaecologist” and “who is responsible if something goes wrong?”) and others being more pragmatic (eg, “Sometimes burglars break into houses, but that didn’t stop us from building houses”). All participants agreed that commercial organizations must not be granted access to data on PWPs. Some participants would like to be able to refuse access by certain health care providers.

##### Benefits of Patient Web Portals

Most participants were positive about PWPs (eg, “I can’t think of negative points”), especially in terms of their potential to be used as a reference site and improve transparency. The ability to access the portal from any location, as needed, was also seen as positive. Some participants mentioned that PWPs could reduce errors because medical costs, prescriptions, and test results will be checked by the patient (“Is it correct what was told [during consultation]?”).

##### Drawbacks of a Patient Web Portal

Some participants were afraid that the provided information would be too complicated, that they would receive too much information, or that it would cost the physician too much time. One participant did not want access to a PWP because she thought it would be too complicated for her, even though she wanted more insight into her medical information:

There is much talking about patients, but not always with patients…Most PWPs I have seen are not user-friendly.

Other participants were worried about practical problems, stating that all PWPs should be comparable and all health care providers should be able to work with them, specifically mentioning the potential difficulties in merging medical information from different health care providers. Several felt that merging the information in a PWP could enhance communication between health care professionals and allow GPs to receive information from the hospital faster (eg, “It would be nice […] if I don’t have to tell my story every time”).

##### Experiences of Patients With a Patient Web Portal

Two participants had experienced medical errors and felt they could have been prevented if they had access to a PWP. One patient told that he could have prevented a wrong surgery (left vs right shoulder) and other patients experienced that their health care provider forgot to notify them about deviating lab results. If they had had access to a PWP, they could have prevented this. Patients can use the information from the patient portal to check whether they have correctly understood the information provided during the consultation.

##### Paying for a Patient Web Portal

Our participants did not want to pay for the PWP because they consider it part of routine care that should be covered by health insurance: “If you have to pay, less people will be interested.” They suggested examining whether a portal could save costs through improved disease control.

#### Patients’ Needs Regarding a Patient Web Portal

##### Preferred Device to Access the Patient Web Portal

It was agreed that the PWP should be assessable by a computer, and possibly by tablet, but that smartphone access may be unsuitable because the screen is too small.

##### Easy to Use and Understandable Language

The PWP should be clear, easy to use, and provide easily understood medical information. All participants agreed that there should be clear instructions about how to use the portal (eg, through an instruction video with access to an information and communications technology helpdesk):

The website must be clear, so that you know where to click and when.

##### Preferred Features in a Patient Web Portal

As PWPs were unfamiliar to most participants, they had difficulties thinking of useful features. To assist them, we screened videos with examples of common PWPs used by Dutch hospitals. The self-management apps that the participants preferred, together with their main comments, are summarized in Multimedia Appendix 1 (summary of the preferred self-management features for patient portal).

##### Ways to Log In

Most participants have experienced DigID, which is a service provided by the Dutch government to provide secure log-in to government websites or medical insurance companies. As DigID was in the news because of fraud at the time of the focus groups sessions, most were worried about the safety of this system (eg, “It is like Big Brother”). They also wanted certainty that their medical records would be separate from those maintained by other governmental organizations or health care insurance companies. Furthermore, it was stated that DigID could be difficult to use, so other log-in options were discussed (eg, short message service; password; finger scans; face recognition; iris scan; or a specific card, like a bank card).

### Insurance Companies

A major concern about medical privacy revolved around access by health insurance companies. Most expressed negative feelings regarding these companies and were fearful that their insurance options could be negatively affected if they were involved in the PWP (eg, “If they [insurance companies] receive information, they can exclude you from certain insurance packages”). Therefore, they did not want medical data to be accessible by insurance companies.

## Discussion

### Principal Findings

It was clear that an essential requirement of a PWP was Web-based access to medical records with an explanation of their meaning. Indeed, despite significant variations in internet experience, and despite the possibility of anxiety because of a lack of understanding, most participants still wanted Web-based access to their medical results. Most also wanted access to crude laboratory results, though they accepted the need for information to be presented at a level that they could understand. Overall, there was some consensus that a PWP should contain test results, a medication overview, information for others, links to reliable websites and a patient forum, and provide the ability to book and participate in Web-based appointments. Tools for disease monitoring and the provision of reliable lifestyle information would also be appreciated by some, but most would not use these options. These findings can help professionals to facilitate the development of a PWP to the needs of patients with asthma and COPD.

### Comparison With Current Literature

Although participants in our focus groups were positive about PWPs, health care providers do not always feel the same. Physicians in Sweden, for example, were afraid that patients would not understand the context of records and might become anxious, which would increase their workload [47]. Moreover, a PWP can be seen as a threat if physicians feel that patients are monitoring their work [43]. In contrast, other studies have shown that PWPs can be more convenient for physicians, not only by saving time on the telephone but also by introducing organizational efficiencies and reduced workflow through greater patient involvement [48].

### Costs and Security

Participants thought that the costs for the PWP should be covered by their health care insurance, even though existing health care systems are not designed to cover Web-based programs [28]. PWPs might reduce unscheduled health care visits [43], although more research is needed to evaluate the cost-effectiveness of PWPs [17]. It was interesting that security was not a major concern, despite the recognition that issues concerning safety and privacy were potential barriers to PWP use [31]. The government-developed DigID log-in method used in the Netherlands was viewed negatively because it was in the news related to fraud. This will have influenced the opinions.

### Patient Web Portal Users

Opinions about internet and PWP use varied among the focus groups, but were consistent with existing research; patients with least internet experience were least likely to want to use a PWP. Research shows that portal users are more experienced with the internet [7], are typically younger and female [22], and have better knowledge of their disease [41]. Developers can facilitate PWP use among the elderly and those with low socioeconomic status by providing explanations in plain language. This might include audio messages for laboratory results [31], videos [27] or Web-based tutorials [31] about how to use the PWP, or pictures for people who have difficulties reading [39]. PWPs should, therefore, be customized to these needs of users [48], with continued efforts to listen to users and make further adjustments over time [7].

### Options That Should be Available in a Patient Web Portal

Participants generally agreed that PWPs should provide access to medical records, a medication overview, and reliable information, which is consistent with previous research, indicating that patients wanted to view laboratory results, refill medications, make appointments, and communicate with their doctor [7]. Several researchers have evaluated the effect of Web-based access of patients on disease control. At present, there are doubts as to whether providing patients direct access to crude numeric laboratory results is wise, not least because it can create confusion or anxiety if patients lack the expertise to interpret their results [49]. One solution might be to incorporate a delay before Web-based publishing to allow physicians time to discuss results with patients. On balance, however, the existing literature is inadequate to allow us to conclude whether laboratory results should be provided immediately after a delay [15].

Links to external websites were considered an important feature because of difficulties finding reliable websites. It might also be useful to incorporate links to self-care information and exacerbation prevention [25]. A reliable website provided by Dutch GPs with Web-based health information for patients was related to a reduction in health care visits, with the average age of the participants being 40.2 (SD 22.9) years [50]. However, these websites can be difficult to understand [39], and developers must be critical when selecting external websites.

All participants were divided about the role of communication with their health care provider. Research has shown that Web-based consultations can be cost-effective for patients by reducing the need to attend in person, though this is often at the expense of insufficient information needed for assessment [51]. It is also unclear what effect secured messaging has on regular face-to-face contact, with some studies showing that it can reduce the numbers of outpatient visits, telephone calls, and emails [28], and others showing the opposite. However, it is generally agreed that patients and providers should use secured messaging specifically for questions that are not urgent [52]. On balance, it appears that Web-based visits do not change the frequency of face-to-face visits [51], with most recognizing that a PWP is no substitute for such contact [31]. If messaging is properly organized in a PWP and inboxes are monitored [9], this service can develop to include advice and encouragement messages and may help increase the usefulness of the system [31].

Finally, the participants in this study were less enthusiastic about lifestyle support options. This is consistent with other research that shows that patients consider laboratory results and treatment goals as most important, with lifestyle support less relevant [41].

### Barriers and Facilitators to Patient Web Portal Use

During the focus group sessions, participants repeatedly said that information needs to be understandable and the portal should be “easy to use.” Research consistently indicates that, regardless of the educational level [10], patients prefer information that is presented in lay language [12]. Smart phrases and standardized text could facilitate this change to lay language. Moreover, PWPs should only contain essential information [12], and developers should consider that patients with low health literacy will have particular difficulty interpreting numbers and risk estimations [30]. The information should also be available in a printable format because patients perceive Web-based information as less trustworthy than printed information [53].

Although PWP use is influenced by personal factors, provider endorsement, and usability [54], the latter is the most important barrier. As patients with COPD often have low socioeconomic and educational status, it is essential that navigating through the different pages is easy and the interface is predictable. Language should be comprehensible and simple. Textual material can be supported by multimedia to enhance the understanding. For example, pictures or videos can help to reach patients who have reading difficulties. Text-to-speech engines are promising and can support patient with reading difficulties [27]. Developers can be supported by automated algorithms that link medical jargon to lay language [55].

In addition, it may be relevant to address patient expectations and take their habits and intentions into account [56]. A pilot of a proposed PWP would be helpful, especially if a patient’s own doctor stresses the potential benefits [11,32,41,56]. Healthcare providers will also need to establish specific training activities so that health care professionals can learn how to work with the portal [11]. Finally, for successful implementation, PWPs should be supported by technicians who can help with technical problems [14,44].

### Effect of Patient Web Portals on Patient-Physician Communication

The patient-physician relationship could change if patients become better informed about their disease after introducing a PWP. Many of our patients felt that communication could become more equal if there were less of a knowledge differential. This is consistent with the results of a study in which patient-reported outcome measures were shown to produce better communication and decision-making between patients and health care professionals [9]. However, no study has specifically looked at the effect of PWPs on communication, and some researchers have argued that physicians can be worried that time spent on the PWP will reduce time available for face-to-face patient contact and that physicians can feel a loss of control if the patient is more engaged in their care [47]. For example, the implementation of a PWP for radiology results led to worries among radiologists [48]. It will be important to secure the involvement of clinicians and address their concerns if a PWP is to be successfully implemented [27].

### Strengths and Limitations

In this paper, we presented an overview of 9 focus group sessions with patients who had asthma and COPD. The strength of this study is that these discussions were open, with 3 groups meeting 3 times at weekly intervals. Therefore, participants got to know each other and shared personal thoughts and emotions with the group. However, selection bias might have occurred because participants might have been more interested in PWPs compared with the general population. For example, participants were included through the Lung Foundation, which suggests that they already had a degree of involvement in their illnesses. Internet experience also varied significantly, and although most were regular internet users, we tried to overcome this issue by stressing that we welcomed participants without internet experience and from areas where the average socioeconomic status was low. Thus, we improved the breadth of internet experience in our groups.

Another drawback of this study is that participants did not use real PWPs but were discussing hypothetical portals. This is important because the intention to use the PWP might differ from the actual use. To improve this issue, we presented videos and screenshots of a variety of example PWPs; for example, we showed examples of PWPs when our participants had difficulties thinking of useful apps. An unintended but inevitable consequence of this is that it was difficult to present suggestions without leading patients. We militated against this by presenting as broad a range of options as possible and allowing participants to choose their preferences. Nevertheless, further investigation with real access to a PWP is needed to understand how patients use portals.

Finally, this was a qualitative study with a small sample that was limited to patients with at least respiratory disorders, and possibly many of them had other morbidities as well [57]; the results cannot be generalized to all patients with asthma and COPD. However, this was not the aim of this qualitative study. Before this research, we did not have a real understanding of the opinions of patients with asthma and COPD regarding a PWP, so we started this study with an open mind and allowed patients to share their opinions freely. This would not have been possible in a quantitative study.

### Conclusions

In general, the participants of this study were positive about PWPs and considered them a logical step in health care development, consistent with the facts that patients are better educated and that most households have access to the internet nowadays. Given that patients are also more assertive and better informed about their disease, PWPs can support them and their interaction with health care professionals. Our participants provided very practical suggestions for implementation in current and future PWPs. The next step should be to develop a PWP with these suggestions in mind and to test whether the portal meets the needs of both patients and health care providers. Future studies can evaluate options for users with asthma and COPD to optimize the PWP.

## Appendix

Multimedia Appendix 1 Opinions about the self-management features.

## Abbreviations

ACO: asthma-COPD overlap
COPD: chronic obstructive pulmonary disease
GP: general practitioner
PWP: patient Web portal
