# Supplementary material for: Assessing the Needs and Perspectives of Patients With Asthma and Chronic Obstructive Pulmonary Disease on Patient Web Portals: Focus Group Study
Source: JMIR Form Res. 2018 Nov 22;2(2):e22. doi: 10.2196/formative.8822 (PMC6334706; doi:10.2196/formative.8822)
Supplement: Multimedia Appendix 1 [file formative_v2i2e22_app1.pdf]

Preferred self-management features	Comments	Illustrated by a citation
Disease monitoring	• Most patients did not want self-monitoring<br>• Some were worried about the reliability of physiological home measurements<br>• Disease monitoring through with for example online questionnaire use would only be useful if the results will were discussed with healthcare professionals<br>• Data presentation on a graph could be helpful (e.g., using baseline peak flows to detect deviations that guide contact with the doctor).<br>• Adding details of an emergency plan in the PWP was advocated.<br>• All agreed that the PWP should not substitute face-to-face contact.	• *"I know how I feel"*<br>• *"I think a questionnaire is more reliable compared to these [physiological] measurements"*
Making appointments online	• An online facility to make appointments was popular<br>• This is unsuitable for assessing emergencies.<br>• This should be reserved for scheduled assessments.	• *"Like for a mechanical check-up for my car"*
Peer contact	• Half of the patients would like to have face-to-face contact with other patients<br>• only a minority would like to use an online	• *"…would prefer to go to locations where [they] can meet peers to have face-to-face contact"*

	patients forum because patients complain too much<br>• Links to recommended patient forums could be provided.	• *"In a patient forum [it] is like a competition: who is the sickest of all"*
Information for others to reduce social implications	• Concise reference information to increase understanding for employers, friends, and family.<br>• Greater use of pictures (easier to understand than text).<br>• Lifestyle applications (e.g., smoking cessation or weight loss).	
Medication overview	• A medication overview with information about how and when to take medications including a facility to reorder prescriptions.<br>• Some wanted to monitor their medication use, but others did not<br>• A medication overview could allow changes in medication to be shared among healthcare professionals.<br>• Educational videos showing correct inhaler technique.	• *"Which one [inhaler] should I take first?"*<br>• *"when I feel ill and tired, I won't sit behind the computer!"*
Links to reliable websites	• Links were requested to guide users to appropriate websites (e.g., from the Dutch Lung Foundation).<br>• Including information about regulations regarding	

	their illness and financial issues.<br>• Sources of self-management devices or specific ergonomic materials.	
Information about inhaler technique	• Information about how to take you medication was mentioned by some patients.	• “If I would see this [inhaler technique in PWP] I would welcome that.”
